# Mathematical model describing erythrocyte sedimentation rate. Implications for blood viscosity changes in traumatic shock and crush syndrome

**DOI:** 10.1186/1475-925X-4-24

**Published:** 2005-04-04

**Authors:** Rovshan M Ismailov, Nikolai A Shevchuk, Higmat Khusanov

**Affiliations:** 1 Department of Epidemiology, Graduate School of Public Health, University of Pittsburgh, Pittsburgh, PA 15213, USA; 2 Center for Cancer and Immunology Research, Children's Research Institute, Washington, DC, USA; 3 Institute for Biomedical Sciences/Program in Molecular and Cellular Oncology, Washington, DC, USA; 4 Institute of Mechanics and Seismic Stability of Structures, Academy of Science of Uzbekistan, Tashkent, Uzbekistan

## Abstract

**Background:**

The erythrocyte sedimentation rate (ESR) is a simple and inexpensive laboratory test, which is widespread in clinical practice, for assessing the inflammatory or acute response. This work addresses the theoretical and experimental investigation of sedimentation a single and multiple particles in homogeneous and heterogeneous (multiphase) medium, as it relates to their internal structure (aggregation of solid or deformed particles).

**Methods:**

The equation system has been solved numerically. To choose finite analogs of derivatives we used the schemes of directional differences.

**Results:**

(1) Our model takes into account the influence of the vessel wall on group aggregation of particles in tubes as well as the effects of rotation of particles, the constraint coefficient, and viscosity of a mixture as a function of the volume fraction. (2) This model can describe ESR as a function of the velocity of adhesion of erythrocytes; (3) Determination of the ESR is best conducted at certain time intervals, i.e. in a series of periods not exceeding 5 minutes each; (4) Differential diagnosis of various diseases by means of ESR should be performed using the aforementioned timed measurement of ESR; (5) An increase in blood viscosity during trauma results from an increase in rouleaux formation and the time-course method of ESR will be useful in patients with trauma, in particular, with traumatic shock and crush syndrome.

**Conclusion:**

The mathematical model created in this study used the most fundamental differential equations that have ever been derived to estimate ESR. It may further our understanding of its complex mechanism.

## Introduction

The erythrocyte sedimentation rate (ESR) is a simple and inexpensive laboratory test that is widespread in clinical practice for assessing the inflammatory or acute response [1]. The ESR has also been found to be of clinical significance in the follow-up and prognosis of non-inflammatory conditions, such as prostate cancer [2], coronary artery disease [3], and stroke [4]. In addition, the ESR can be used in the diagnosis of inflammatory conditions [4,5] as well as in the prognosis of non-inflammatory conditions [6]. Some examples of recent applications of the ESR may include sickle cell disease and bacterial otitis media [7,8]. The ESR has been shown to be elevated in 55% of patients with otitis media [7]. Those with elevated ESR have been shown to have a much higher risk for recurrence [7]. In sickle cell anemia, the ESR is usually low in the absence of a painful crisis [8]. A low ESR is an intrinsic property of the sickle red blood cell rheology [9,10]. Certainly, the ESR is only one parameter among others that a clinician can use in the diagnosis and follow up of the above diseases.

Sedimentation of particles, in particular erythrocytes, in a Newtonian fluid (plasma), has been studied by many investigators based on the theory and, therefore, model of interpenetrating motion of two-phase medium that take into account aggregation of erythrocytes [11,12]. We aimed to investigate group precipitation of particles, such as erythrocytes, in a two-phase medium (plasma), both theoretically and experimentally. We added the influence of the vessel wall on group precipitation of particles in tubes, as well as the effects of rotation of particles, the constraint coefficient, and viscosity of a mixture as a function of the volume fraction. The theory has also taken into consideration certain experimental coefficients such as: the coefficient of interaction between the fluid and particles, the aggregation coefficient, the constraint coefficient of phases, the coefficient of viscosity of the mixture, and the coefficient of rotation of a particle. The equation system has been solved numerically. To choose finite analogs of derivatives we used the schemes of directional differences.

## Methods

Stokes[13] was the first who derived an equation for non-steady-state flow when he was linearizing the equation of motion of a viscous incompressible fluid. In that work, Stokes developed a theory of resistance for a falling spherical body. The relationship that he derived is called Stokes' formula:

*F *= 6*πμ aV*,     (1)

Where *μ *represents viscosity of the fluid, *a *– the radius of the sphere, *V *velocity of the fall, and *F *resistance force.

Albert Einstein investigated the disturbances caused by a particle suspended in a flow with a constant velocity gradient [13,14]. He developed a theory of resistance to shear for a suspension of small spherical particles in a continuous fluid medium. He proved theoretically that an increase in viscosity of a fluid carrying solid particles is connected to the volume fraction of the particles via a proportionality coefficient:

*μ *= *μ*_0 _(1 + 2.5 *f*_2_),     (2)

where *μ*_0 _represents viscosity of the fluid, and *f_2 _*concentration of particles.

Up to now, the Einstein formula of viscosity of suspension has been the foundation for most theories describing behavior of a suspension in a shear flow [13,14]. Most studies deal with precipitation of a single particle or multiple diluted particles in a Newtonian viscous fluid and offer various corrective parameters for the Stokes formula [13,14]. For instance, Ozeen [13,14] deduced an approximate solution of equations for a flow of spheres that served as a basis for the formula



where *N*_Re _= *aVρ*/*μ *is the Reynolds number.

Subsequently, some problems related to non-Newtonian behavior of fluids [13,14] were also investigated. Casuell and Schwarz applied Ericson and Rivlin's model for a slow flow of a non-Newtonian fluid [13,14]. Applying the method of jointed expansions that uses corresponding non-Newtonian terms for both "internal" and "external" expansions to the formula of spherical flow, they derived the following formula:



where *φ *is a compound expression dependent on non-Newtonian parameters that are constant for any given fluid under isothermal conditions. Lesli also investigated a slow spherical flow, using the Oldroid model [13,14] for non-Newtonian conditions. He concluded that the non-Newtonian term is proportional to *V*^3^.

The above studies refer to the precipitation or flow of a single spherical particle. However, concentrated mixtures with a large number of particles interacting during precipitation or in a flow are affected not only by the Stokes force shown in equations (1), (3), and (4), but also by other forces given in equation [14]:





where  is a buoyancy force,  – frictional force or Stokes force resulting from the viscous force during interaction between phases, determined by the difference between velocities (slippage) *u*_1 _- *u*_2_, size – a, by the quantity and shape of inclusions, as well as by the physical properties of phases; *p*-pressure difference, *χ*(*m*) – coefficient of constraint, *ρ_1 _*and *ρ_2 _*are densities of the first and the second phase and are determined by the theory of multiphase medium as reduced densities, *K*^(*μ*) ^– coefficient of phase interaction, *μ_1 _*and *μ_2 _*– viscosity of the first and second phase (where first and second phases correspond to plasma and erythrocytes respectively), *f_2 _*– concentration of the second phase,  – force related to the effect of "added masses" and caused by the accelerated motion of inclusions (particles) relative to the carrying (viscous fluid) medium, when disturbances arise at a distance on the order of the size of particles;  – force of additional effect on particles, due to gradients in the area of average velocities in the liquid phase (the Magnus or Zhukovskiy force). This force is a result of the difference between the densities of phases and the difference between pressures on the opposite sides of a flowing particle.

The influence of the shape of particles, their multiplicity, and some other factors in the expressions for forces , ,  are taken into account in coefficients *K*^(*μ*)^, *χ*^(*m*)^, *χ*^(*r*)^. As a first approximation, the data on precipitation of a single particle or of a body of a corresponding shape can be applied here, disregarding the direct influence of particles on each other, but taking into account the constraint of the flow of fluid around particles that is caused by the multiplicity of particles [11,14,15].

In order to account for the Magnus effect connected to , it is necessary to take into account the rotation of particles, and in the general case, also the corresponding kinetic parameter *ω*_2_, which represents the velocity of rotation that is independent of the field *u*_2 _[14]:



where  – coefficient accounting for the shape of particles ( = 4*π*/3 for spherical particles with radius a). In addition to convection, the value of *N *(the number of particles per unit volume) may change, due to the processes of crushing, adhesion, aggregation of particles, and formation of new ones that are defined by value *ψ *in the equation [14]:



In case of the absence of crushing, adhesion, and aggregation of particles or formation of new ones, i.e. when *ψ *= 0, as well as under the condition of non-compressibility of the material (of particles), that is *ρ*_2 _= constant *α *then  is a constant. Moreover, the equation of conservation of mass of the second phase[14] leads to . And *N*, number of particles per unit volume, can be expressed as [14]:



or [14]

∂*N*/∂*t *+ ∇*Nu*_2 _= 0.     (9)

It should be noted that in the case of a moderate volume fraction of particles in a fluid, the mixture of two incompressible phases cannot be considered in terms of Navier – Stokes' equations, even in the absence of relative rotary and radial motion [14]. This is because viscosity of suspensions and emulsions is determined using viscosity-meters based on the measurement of a mass consumption of mixture Q through a tube, with diameter d and length L, dependent on pressure ∇P [16-18]. If a Poiseuile flow of a Newtonian fluid is the case, then a linear connection between Q and ∇P holds true. It follows, then, that with low concentrations, when *f_2 _*< 0.05, the resulting values of *μ*_*ef *_agree with the Einstein formula (2), but when *f_2 _*> 0.05 then values *μ*_*ef *_exceed values obtained from the equation [14]:



Additionally, there is considerable variation among different authors and among different combinations of phases. This variation apparently reflects the non-Newtonian nature of concentrated viscous disperse mixtures and insufficiency of values *ρ *and *μ *for determining their mechanical properties. In this regard, laboratory experiments have to be carried out for each mixture and real devices over a range of operating parameters to determine the loss of pressure when applying different rheological models, in particular, the model of a viscous fluid with an effective coefficient of viscosity.

One should keep in mind that when *f*_2 _≥ 0.1, in addition to the shape and size of solid particles, their material may also have an effect on effective viscosity and other rheological characteristics of the mixture. Apparently, this is due to irregular distribution of particles, collision of particles with each other, and solid walls [16-18].

The above considerations lead to an examination of group precipitation of particles, when *f*_2 _≥ 0.1 in the interpenetrating model of two- or multiphase medium. The first report of such an approach was presented in [11], where researchers used the theory of interpenetrating motion of two-phase mediums, taking into account the aggregation of particles, in this case erythrocytes, in a fluid layer limited by a free border x = L from the top and a solid border x = 0. In those studies, precipitation of particles in a fluid was considered based on the following assumptions: the motion of particles in a fluid is taking place in the strictly vertical direction under the influence of gravity; the inertial force is negligibly small; viscosity appears only during interaction between particles and the fluid surrounding them; spontaneous disintegration is absent; the column of sedimentation of particles is divided into two main zones: clean fluid and settling particles. The influence of the wall and interaction between particles were ignored. Studies [19,20] showed that the radius of the tube has an effect on sedimentation of particles, in particular, erythrocytes. From this it follows that the group precipitation of particles, such as erythrocytes, depends appreciably on border conditions, in this case on conditions of adhesion and impact of particles on the vessel wall [19,20].

In addition, in most studies the rotation of particles caused by force  was not taken into account, and neither were the force of interaction between adjacent particles or the force  arising from accelerated movement of particles. Thus, in the present work, we examine the group precipitation of particles in a liquid mixture with forces  and , as well determination of the constraint coefficient and of viscosity of the mixture *μ*_*m *_as functions of the volume fraction of particles. We also describe the effect of the vessel wall on the process of group precipitation of particles in a multiphase medium.

### Determination of the coefficient of constraint and viscosity of a mixture as a function of the volume fraction of particles

Problems of constrained precipitation of solid and deformed particles in a fluid are the principal part of the theory of two-phase flows and are of great importance for such hydrodynamic processes as: mass of exchange, separation by gravity, oil refining, and precipitation of erythrocytes in blood [11,12,16-26].

Studies in this field usually deal with high- and low-concentration mixtures. The laws of constrained precipitation of moderately concentrated mixtures have not been well investigated. The foundation of precipitation of a single particle in an infinite fluid is the Stokes law. According to that law, the frictional force arising from the motion of spherical particles with diameter d and velocity V in a medium of viscosity *μ *is expressed by formula



The gravitational force acting on a particle depends on its specific gravity, that is



where *ρ*_1_;*ρ*_2_;*g *– the density of a fluid, density of the particle, and acceleration of gravity respectively.

 – buoyancy force, and, in (11)

 – frictional force.

Due to force  the particle accelerates its motion. Aside from gravitational force, the particle is affected by frictional force directed oppositely; its value increases with the increasing velocity, according to the Stokes law. This means force  is canceled out by gravitational force . Therefore, the movement proceeds with constant velocity *V*_*c *_that is determined by the equations (11) and (12):



Sometimes one has to deal with precipitation of a large number of particles in concentrated mixtures. Formulae for the velocity of precipitation of particles as a function of the concentration and velocity of a single particle in an infinite fluid can be derived using some statements of the interpenetrating model [14] and the Euler equation [14]. From these formulas, an equation for low concentrations of particles can be easily deduced; the equation is consistent with the experimental data [27]. Let us assume that in volume V there are two phases with different values of specific gravity. Then particles with greater specific gravity start moving down canals that are being formed; this holds true for the displacing phase, i.e. the process of mutual penetration is taking place.

Let us determine the hydraulic radius of a canal as



If the canal is cylindrical then



From (14) and (15) the effective diameter of the canal is equal to:



where S – cross-section area of the canal, P – perimeter of the canal. Multiplying both numerator and denominator by diameter *d *(in this case *d *corresponds to the diameter of a single erythrocyte) and by volume V and also taking into account [21] that external concentration equals volume concentration, it follows:



where *S*_*u *_– surface of the particles; *f*_1 _– volume fraction of the first phase. But:



(where *N *– number of particles per unit of volume equal to ), therefore (16), (17):



(where  is a coefficient of shape for spherical particles).

For particles of irregular shape:



( – form coefficient).

We can divide the continuity equation:

*V*_1_*S *= *V*_1*i*_*S*_1_

by *S*, and multiply both numerator and denominator by diameter *d *(in this case *d *corresponds to the diameter of a single erythrocyte) and by volume V and also taking into account [21] that external concentration equals volume concentration. Then it follows:

*V*_1 _= *f*_1_*V*_1*i *_    (19)

The flow of fluid between particles by analogy with the flow of fluid in tubes can be expressed as criterion equations [23]:



or [23]:



In the processes of precipitation when concentration of inclusions is significant and sizes of particles are small, and in the processes of industrial filtration as well, a laminar flow is very often observed, for which m = -1, n = 1. Then [23]:



If one takes into account (18) and (19), the last equation can be expressed as Coseni Carman for constrained precipitation in a laminar flow:



Let's divide equation (23) by the number of particles per unit of volume and derive the resistance force of the fluid acting on one particle as:



(*χ *– resistance coefficient for precipitation of a set of particles).

It is known that the resistance force experienced by a particle during precipitation in a fluid is:



Since for particles suspended in a fluid:

F* = *F*_*c*_

Then from (24) and (25) it follows:



where *β *– the ratio of velocity to hydraulic size;

*χ*_*c *_– resistance coefficient for precipitation of a single particle (25) in an infinite fluid. From (24) when *f*_1 _→ 1 it follows:



when values of Reynolds numbers are small:



Therefore, it can be supposed that the basic mechanism is:



From the equations (26) and (27), we can derive:



where



According to the Stokes law, in a laminar flow we have:



figuring the last equation into equation (30), we can derive



In accordance with our experimental data when Reynolds numbers are small, constant C = 5.

Equation (29) when *f*_1 _→ 1 can be expressed as

*β *= 1 - *cf*_2_.     (30)

**Figure 2:**
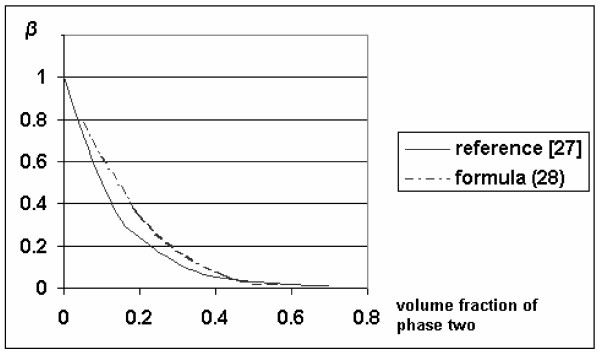
The relative velocity of sedimentation as a function of the concentration of particles. Horizontal axis: volume fraction of phase two; vertical axis: *β*, a change in the relative velocity.

The difference between the coefficients of equation (30) and the coefficients calculated for small concentrations [22] is the factor of 0.34. If we do not ignore the fraction of velocity resulting from the proximity of two spheres, which is true for small concentrations of inclusions, then equation (30) coincides with the Bachelor formula [22]. The change in the velocity of particles depends on the concentration calculated from (29); a wide range of concentrations is given in Figure 2.

From the equation of equilibrium of forces, taking into account the coefficient of constraint, it follows (13) [14]:





Based on (31) and (32) we have:



or taking into account (29)



(*φ*_*f *_– coefficient describing constrained precipitation).

Assuming precipitation of a particle in suspension with viscosity *μ*_*m*_, density *ρ*_*m*_, we can express the equilibrium equation [21] as



*ρ*_*m *_= *f*_1_*ρ *_1*i *_+ *f*_2 _*ρ*_2*i*_.     (36)

Allowing for (32) and *V*_1 _= 0, it follows



and from (28) we can obtain



**Figure 1:**
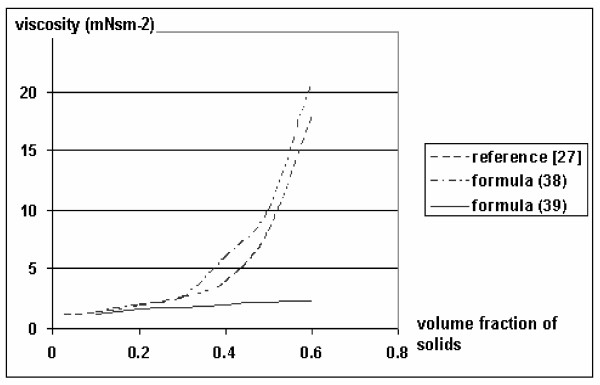
The dependence of a change in relative viscosity on the concentration of particles. Horizontal axis: volume fraction of solids; vertical axis: viscosity (mNsm^-2^)

When *f*_1 _→ 1, but C = 2.5, we have the Einstein formula (10):



From the calculation given in Figure 1, it follows that formula (38) agrees with experimental data (up to *f_2 _*= 0.5, when C = 2.5) obtained in a previous study [27] on the change of viscosity of suspension for a wide range of fluids, sizes, and materials of solid discrete particles.

The above dependence of relative velocity and viscosity of the mixture on volume fraction agrees with the experimental data [27]. Let us examine formulae (33) and (34), and consider the possibility of determining an analytic calculation formula for the constraint coefficient and effective viscosity, and their dependence on the concentration of particles. In (33) and (34) the formula for the relative velocity of the precipitation of particles has been derived as:



where *V*_2 _– velocity of constrained sedimentation of disperse particles;

*V*_*c *_– velocity of sedimentation of a single particle (*f*_2 _≈ 0) in a motionless fluid (*V*_1 _= 0);

*f*_2 _– volume fraction of disperse phase *f_1 _*+ *f_2 _*= 1;

c – dimensionless numerical coefficient.

As is known[14], from the equilibrium equation of a two-phase medium, allowing for constraint motion of particles



We can derive an equilibrium equation of gravitational force, buoyancy force, and Stokes force acting on the disperse phase as:



The parameters correspond to the parameters in the study [14]. The second equation (42) describes a rest condition of the mixture; that is, the motion of dispersed particles in one direction accompanies the motion of fluid in the opposite direction.

From the equation (42) for precipitation of a single particle (*f*_0 _≈ 0) in (*φ*_*j *_= 0) in the at-rest mixture, we can derive the Stokes formula, however the velocity of constrained precipitation is:



From the equations (40) and (43), the constraint coefficient can be determined as:



From the analysis of the experimental data for small volume fractions (*f_2 _*< 0.005), it follows that there are three types of functions [14] for the determination of a constraint coefficient corresponding to different types fluids in which spherical particles are precipitating:







Comparing equations (44) – (47), we can conclude that formula (44) deduced from [28] is similar to formula (46).

When  and *c *≈ 5 ÷ 6; that is, under conditions (44), a change of constraint coefficient is described, depending on the volume fraction of randomly dispersed particles. As described previously [14], when the concentration of the disperse phase in a mixture increases, numerical values of volume and shear viscosity start to differ from each other, and the motion of the mixture cannot be described by Navier – Stokes equations [14].

When concentrations (*f_2 _*< 0.005) and values of viscosity agree with the Einstein formula, and when concentrations determined in the laboratory are high, then the viscosities considerably exceed the values derived from the formula for moderate and concentrated viscous disperse mixtures of non-Newtonian nature. In addition, the rheological parameters are insufficient for the determination of the mechanical properties of those moving mixtures.

In particular, one of the models of viscous fluid is a model taking into account the effective coefficient of viscosity, as an additional rheology parameter. Moreover, it is assumed that the effective coefficient of viscosity (viscosity of a mixture) reflects not only a shape and size of solid particles, but also their material and irregularity of their center of gravity.

Following the approach described in[14] for precipitation of particles in a fluid with viscosity *μ*_*m*_, we can derive the following equality



Using equation (48) and allowing for equation (43) [28], it follows that

*μ*_*m *_= *φ*_*f *_*μ*_1 _    (49)

We can also substitute equation (44) into equation (49), and from the equations (45)–(47) and (49) we can derive [28]



We can summarize here: equation (45) has been derived from the consideration of the cell model for uniform distribution of particles; equation (46) deals with the random allocation of particles; and equation (47) is the case of significant formation of clumps and alignment of particles in chains [14]. Therefore, equations (50) allow one to determine viscosity of a mixture for all three types of distribution of particles. Thus, the formulae for calculation of the constraint coefficient and viscosity of a mixture as functions of the volume fraction of the disperse phase have been fully described.

### Forces in a two-phase medium that affect group precipitation, in particular, precipitation of erythrocytes in blood

As follows from the above, there can be four basic interacting forces when particles are precipitating in a mixture:

1. The buoyancy force occurring due to the difference between densities of phases;

2. The frictional force or Stokes force; it results from interaction between the two phases;

3. The force of "added masses" arising from either accelerated or constrained motion of particles;

4. The force of additional effect on particles which is due to gradients in the area of average velocities in the liquid phase (the Magnus or Zhukovski force).

These forces should be taken into consideration when creating a mathematical model of group precipitation in a multiphase medium.

The first force, buoyancy force, is defined by formula:



where *f*_2 _– volume fraction of the second phase (particles).

Δ*P *– gradient of pressure, or the difference of densities of phases.

The second force, frictional force, or Stokes force, is defined by the difference of velocities (slippage) |*V*_1 _- *V*_2_|, the size, quantity and shape of inclusions, as well as physical properties of the phases. From [11] we can write down the following equation for the frictional force (5), (6):



Instead of viscosity of plasma *μ*_1_*φ*(*f*_2_) we can use the viscosity of the mixture determined in formula (50)







Expression (53) has been derived while applying the cell model for uniform distribution of particles, expression (54) deals with irregular distribution of particles, and expression (55) is the case of significant formation of aggregations and alignment of particles in chains. Therefore, from the equations (54) and (55), one can determine the viscosity of the mixture for all three types of distribution of particles in a fluid. Instead of function *ψ*(*f*_2_) from [11] we can use .

The third force: the force is related to the action of "added masses," i.e. this force arises from either constrained or accelerated motion of particles:



where constraint coefficient *χ*(*m*) from formulae (44), (45) and (46) can be defined as [14]:







Horizontal movement of particles under the influence of gravitational force can be explained, if one considers the flow of precipitating particles. In a homogeneous mixture with a zero average flow rate [24],

*f*_1_*V*_1 _+ *f*_2_*V*_2 _= 0     (60)

Which means that in each point, the volume flow of the precipitating solid phase accompanies upward motion of the fluid. This phenomenon has been considered in the previous paragraph. The following is the formulae for forces of the additional effect on particles, caused by gradients in the area of average velocities in the liquid phase:



In addition, coagulation or aggregation of multiple particles can take place, which is described by these equations (7), (8), (9) [14]:



Where N – number of units, *ψ *– total velocity of aggregation [14].

*ψ *= *KN*^2^,     (63)

where *k *– aggregation coefficient.

Based on the above, the mathematical model for precipitation of particles in a two-phase medium can be formulated.

### The mathematical model for group precipitation in a two-phase medium (mixture) in a flat tube

Let us consider precipitation of particles in a fluid on the basis of the hydrodynamic theory. Experiments on precipitation of erythrocytes in plasma carried out in cylindrical tubes of various diameters have shown that the radius of the tube significantly affects the process of precipitation [29].

It follows then that group precipitation of particles such as erythrocytes, depends on border conditions, in this case the conditions of adhesion. The small radius of a tube defines the area of velocities of flow of the liquid phase during precipitation of a particle. Therefore, particles experience a differential of pressure from the surrounding fluid. That is why, in this study, the fluid (carrying phase) is considered a viscous phase.

Let us consider precipitation of a particle taking place between vertical parallel walls. We will place X axis between the walls pointing upward, Y axis also parallel and equidistant from the walls but horizontal, and Z axis is horizontal and perpendicular to the walls. Let us assume that the horizontal length of the tube is 2 h, and its height is L. We will also assume that the motion is not taking place along Z axis. This leaves us with two-dimensional movement of particles. Group precipitation of the particles in the mixture can be described mathematically as the motion of two-phase interpenetrating mediums according to the theory of Rakhmatullin [21].

It follows that equations for the two-phase flow in a Cartesian space coordinate system can be expressed as [30]:



for the second phase [30]:



Continuity equations for each phase are expressed as [30]:





and the equation of numerical concentration is then [14]



Assuming that the components of velocity V and W are negligibly small compared to component U, and that the horizontal length of the canal is negligibly small compared to the height, that is, V ≪ U, W ≪ U and *L *≫ 2*h*, we can then estimate some terms and simplify the system of equations ; instead of *U*_1_, *U*_2 _we will use u_1 _and u_2_



where *μ*_*m *_– effective coefficient of viscosity of the mixture that is derived from the formula:



or for specific cases







Where *μ*_1 _– viscosity of plasma.

We can write down the continuity equation as





The equation of numerical concentration then becomes:



where N – number of aggregates, *ψ *– total velocity of aggregate formation.

Three forces determine the force of interaction between phases.



The frictional force can be derived from formula (52)



which takes into account the number of aggregates N. Equation (78) also allows an investigator to take into account the changes in the number of particles per unit of volume, the average distance between them, the radius of particles, volume of the disperse phase, etc. These geometrical parameters have a significant effect on the kinematics of the aggregation processes.

From [11] we can derive the expression for the total velocity of aggregate formation

*φ *= -*KN*^2^.     (79)

From expression (78) when , and based on the formula from [31] we can obtain



here  – coefficient of shape for the particles in question.

Instead of the force of "added masses"  we can use formula (56), namely



where *χ*^(*m*) ^– coefficient of constraint, which is derived from one of the following formulae







The Magnus or Zhukovski force is defined by the expression



Therefore, the closed equation system to solve the problem has been obtained:



Using equations (74) and (75) we can obtain



Since the precipitating particles cannot flow through the bottom of the tube, the following holds true:

*f*_1_*u*_1 _+ *f*_2_*u*_2 _= 0.     (88)

Equation (88) gives us the rest condition of the mixture. Let us use the force of interaction between phases given in [11] to derive the closed equation system. First, it is necessary to formulate initial and border conditions.

At *t *= 0 *u*_1 _= 0, *u*_2 _= 0,     (89)

(*N*_*0 *_is the number of particles in 1 mm^3^, *a *– the radius of a particle)



This problem can be solved numerically. We will use the following initial data:



### Analysis of the experimental and numerical results and their comparison

**Figure 3:**
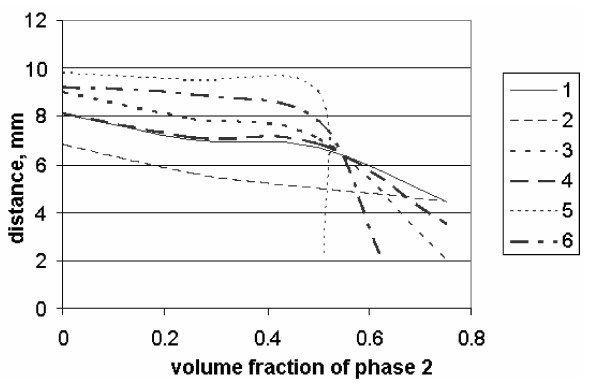
Curves of the change of concentration of the disperse phase along the height of the tube at various moments of time. Curves 1 and 2 when t = 60 min, 3 and 4 when t = 25 min, 5 and 6 when t = 5 min. Curves 1, 3 and 5 correspond to precipitation without border conditions, and 2, 4 and 6 with border conditions. Aggregation coefficient K = 10^-5 ^for all curves. Horizontal axis: concentration of the second phase (*f_2_*); vertical axis: distance (millimeters)

Figure 3 shows the vertical distribution of concentrations of the disperse phase at various time intervals. More precisely, it shows the concentration at various points along X axis at various intervals of time. In figures 3, different curves correspond to the distribution of concentration in the tube after t = 5 min, t = 25 min, t = 60 min from the beginning of the process. It was noted that the change of concentration of the disperse phase along the height of the tube at various intervals of time, corresponds to the decrease of volume fraction of the disperse phase in the upper part and its increase in the lower part of the tube. Moreover, aggregation of particles reinforces the precipitation of erythrocytes.

As one can see, when aggregation coefficient κ is 10^-5 ^the effect is observed, if we compare the curves in figure 3. It should be noted that the wall layer has a considerable effect on precipitation of erythrocytes. Since particles bounce off the walls, the aggregation of particles in the central part of the tube is enhanced. As a result, a big aggregate of particles precipitates faster than a single particle. This effect was described in the theoretical studies, as well as shown experimentally. In order to determine the influence of the vessel wall, we placed the blood of a patient in capillaries of various diameters. During precipitation, erythrocytes descended 18 mm after one hour.

When  they descended 11 mm, but when  by 6 mm.

That means that the influence of the vessel wall on precipitation of erythrocytes is considerable in capillary tubes of lesser diameter. Also, according to figure 3, the curves of precipitation are different at various time intervals.

**Figure 4:**
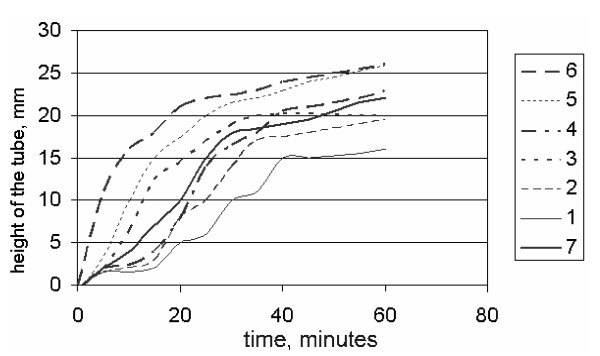
Curves of the change in the borders of separation between fluid and mixture as a function of time, when values of total velocity of aggregation are different. *k *: 1 - *k *= 5·10^-7^; 2 - *k *= 1·10^-6^; 3 - *k *= 5·10^-7^; 4-experimental curve corresponds to rheumatism; 5 - *k *= 1·10^-5^; 6 - *k *= 5·10^-5^. Dotted curves match the results of equations (86), with the border conditions (89) and (90). Horizontal axis: time (minutes); vertical axis: the height of the tube, millimeters.

Figure 4 shows the curve of precipitation of particles as a function of time, for various values of aggregation coefficient K, both in the theoretical (curves 1–3, 5, 6) and experimental studies (curve 4). According to Figure 4, theoretical curves are consistent with the experimental data.

## Discussion

This work addresses the theoretical and experimental investigation of single and group precipitation of particles in homogeneous and heterogeneous (multiphase) medium, as it relates to their internal structure (coagulation or aggregation of solid and/or deformed particles). Such processes cannot be described by means of a single velocity model of a non-Newtonian fluid. Precipitation of particles in a viscous homogeneous relaxing medium and the motion of flow of the fluid are directed oppositely, due to the gravitational force. These processes can be described by means of a two-velocity interpenetrating, two-phase (or multiphase) model.

In general, ESR has a complex mechanism where, on the one hand, an increase in the concentration of erythrocytes leads to a decrease in *β*, the change in the relative velocity of sedimentation (figure 2). On the other hand, such an increase may result in an increase in the quantity of rouleaux. Our mathematical model takes into account the influence of the vessel wall on group precipitation of particles in tubes, and also viscosity of the mixture, constraint, and rotation of particles. This model can also describe ESR, using the coefficient of the velocity of erythrocyte precipitation.

As mentioned earlier, ESR measurement remains the method of choice in evaluating different clinical conditions [5]. In our view, the ESR could also be useful in patients with the traumatic shock and traumatic crush syndrome. In general, conditions such as hemorrhage, trauma, and burns may result in blood viscosity changes [32]. Erythrocyte aggregation caused by trauma, burns, and hemorrhage was described in detail by Knisely [33].

In the 21st century, at the time of high-speed transportation and motor-vehicle accidents, critical states, such as traumatic shock, have become more widespread. Traumatic shock results in generalized vasoconstriction, and therefore, this condition may result in microcirculatory disorders that have been associated with increased blood viscosity [34,35]. Traumatic shock, due to increased blood viscosity, may further decrease the cardiac output [34].

Theoretically, any trauma may result in extensive intravascular erythrocyte aggregation, irrespective of the presence or absence of traumatic shock [36]. However, generalized intravascular blood slowing and stasis, as well as erythrocyte aggregation, is much more severe in the presence of traumatic shock [36]. The analysis of the certain rheological changes caused by the experimental traumatic shock was conducted by Tatarishvili et al [35]. In total, 40 adult male laboratory rats were used, of which 20 were used for experiments and 20 served as controls [35]. Kennon's method was used to produce traumatic shock. Erythrocyte aggregability was measured using the "Georgian technique"[37]. Traumatic shock resulted in significant changes in all investigated blood rheological parameters (erythrocyte aggregability, deformation, and systemic hematocrit). The experimental group of rats showed an increase in erythrocyte aggregability, which was more than 2 times higher compared to control animals. However, the hematocrit and erythrocyte deformability decreased in experimental rats compared to control animals [37]. Despite the small sample size, these results were statistically significant [37]. Tatarishvili et al. concluded that "the blood rheological disorders in the microcirculation related in the present experiments should be considered as a significant factor determining the severity of the damage to the tissues during the development of the traumatic shock" [37].

As mentioned earlier, the ESR measurement might also become a useful diagnostic test in patients with crush syndrome. Disasters, such as earthquakes, are a key source of victims with crush syndrome. Earthquakes in Armenia in 1988 [38] and in Japan in 1995 have caused multiple cases of traumatic crush syndrome. [39] Victims of such earthquakes have been trapped for prolonged periods. The other possibility for the crush injury and crush syndrome is, for example, the severe and transient pressure that occurs when a limb is run over by a heavy vehicle. Finally, crush syndrome may also affect patients with stroke or drug overdose [40]. Such patients could crush a part of the body with their own weight while unconscious. The lower limbs and, less frequently, upper limbs are predominant sites of injury in victims with traumatic crush syndrome. Traumatic crush syndrome caused by a crush injury to the torso has also been described [39]. Rheological changes caused by traumatic crush syndrome have been studied in rats that were exposed to 6 hours of compression of soft tissues on the thigh. Narcotized rats with crush syndrome were shown to have increased blood viscosity. In these animals, total peripheral vascular resistance was shown to correlate well with changes in blood viscosity at different shear rates. [41]. Rats' blood viscosity was predominantly determined by erythrocyte aggregation at low shear. [41] These rats were also divided into two groups: low resistant and high resistant to shock. Low resistant animals exhibited high hematocrit and plasma viscosity and decreased deformability of erythrocytes. On the contrary, blood viscosity increased, independent of shear rates (10 – 300 sec ^-1^). Severe hemoconcentration and blood hyperviscosity developed in all low resistant animals. High resistant rats developed sufficient hemodynamics and oxygen supply to tissues. On the other hand, high resistant rats exhibited less significant hemoconcentration and an increase in blood viscosity [42].

According to our current research, increased ESR in patients with traumatic shock and crush syndrome is due to an increased number of rouleaux. Our understanding of changes in the ESR in traumatic shock or in crush syndrome could be summarized as follows: crush syndrome or traumatic shock (due to hemorrhage) may result in an increase in peripheral resistance[34,43-45]. Increased peripheral resistance may result in a decrease in cardiac output, which subsequently leads to a decrease in blood flow velocity and, thus, a decrease in yield velocity and shear stress[46] A decrease in blood flow velocity, due to trauma, may result in a decrease of erythrocyte adhesiveness within rouleaux [46], since even a relatively high yield velocity (300 m^-1^) did not result in a significant decrease in blood viscosity [41,42]. The damage of particles at different erythrocyte adhesiveness within rouleaux has been studied in the previous research [47]. From those experiments it follows that the damage is proportional to yield velocity [46]. On the other hand, an increased ESR in patients with traumatic shock and crush syndrome is due to an increased number of rouleaux, however, as it has been shown earlier, the quantity of rouleaux is largely dependent on the quantity of erythrocytes [47]. Going back to the beginning of the discussion, those rats that had lower survival (second group) had, according to our hypothesis, a larger quantity of rouleaux formation, due to an initially larger quantity of erythrocytes [41,42].

## Conclusion

The mathematical model created in our study accounts for the influence of the vessel wall on group precipitation of particles in tubes, viscosity of the mixture, constraint and rotation of particles. The determined viscosity of a mixture and constraint of particles depend on volume fraction. In theoretical studies, the difference of curves of precipitation, during the initial and later intervals of time, has been established.

The theoretical curves of precipitation of particles at various values of aggregation coefficient K show that an increase of K increases the velocity of precipitation. Likewise, the presence of the tube wall enhances precipitation of particles. The Magnus force and the force connected to acceleration of particles in a relatively liquid phase do not have a significant effect on the precipitation of particles. From our experimental and theoretical data, we can conclude that the behavior of the curve of velocity of erythrocytes depends on the timing of observations (interval of time is 5 min). Time-course measurements of erythrocyte precipitation more accurately reflect the state of the human body than the known determination of velocity of erythrocytes.

Determination of the ESR should be conducted at certain intervals of time, i.e. in a series of periods not exceeding 5 minutes each. Differential diagnosis of various pathological conditions when ESR is applied can be conducted using the aforementioned timed method of determining ESR. Different pathological states, when this method is used, would have different initial blood viscosity parameters. We have shown that an increase in blood viscosity during trauma results from an increase in rouleaux formation. We have shown that the time-course method of ESR will be relevant in patients with trauma, in particular, with traumatic shock and crush syndrome. Therefore the number of rouleaux is dependent on the initial quantity of erythrocytes and is increasing sharply with the decreasing yield velocity [47] which may result from increased peripheral resistance and low cardiac output from traumatic shock or crush syndrome.

## References

[B1] Saadeh C (1998). The erythrocyte sedimentation rate: old and new clinical applications. South Med J.

[B2] Johansson JE, Sigurdsson T, Holmberg L, Bergstrom R (1992). Erythrocyte sedimentation rate as a tumor marker in human prostatic cancer. An analysis of prognostic factors in 300 population-based consecutive cases. Cancer.

[B3] Gillum RF, Mussolino ME, Makuc DM (1995). Erythrocyte sedimentation rate and coronary heart disease: the NHANES I Epidemiologic Follow-up Study. J Clin Epidemiol.

[B4] Chamorro A, Vila N, Ascaso C, Saiz A, Montalvo J, Alonso P, Tolosa E (1995). Early prediction of stroke severity. Role of the erythrocyte sedimentation rate. Stroke.

[B5] Sox HC, Liang MH (1986). The erythrocyte sedimentation rate. Guidelines for rational use. Ann Intern Med.

[B6] van den Hoogen HM, Koes BW, van Eijk JT, Bouter LM (1995). On the accuracy of history, physical examination, and erythrocyte sedimentation rate in diagnosing low back pain in general practice. A criteria-based review of the literature. Spine.

[B7] Del Beccaro MA, Mendelman PM, Inglis AF, Richardson MA, Duncan NO, Shugerman RP (1992). Acute-phase reactants and acute bacterial otitis media. Am J Dis Child.

[B8] Ballas SK (1995). The sickle cell painful crisis in adults: phases and objective signs. Hemoglobin.

[B9] Akinola NO, Stevens SM, Franklin IM, Nash GB, Stuart J (1992). Rheological changes in the prodromal and established phases of sickle cell vaso-occlusive crisis. Br J Haematol.

[B10] Lawrence C, Fabry ME (1986). Erythrocyte sedimentation rate during steady state and painful crisis in sickle cell anemia. Am J Med.

[B11] Losev ES (1992). A physical model of gravitational erythrocyte sedimentation. Biofizika.

[B12] Normatov TD, Khusanov IN (2001). Determination of constraint coefficients and viscosity of mixture in dependence on volume fraction of particles. Nauka.

[B13] Happel J, Brenner H (1986). Low Reynolds Number Hydrodynamics with Special Applications to Particulate Media.

[B14] Nigmatullin RI (1978). Basic mechanics of multiphase medium.

[B15] Faizulaev DF, Shakirov AA, Abidov S, Khusanov IN (1974). Investigation of precipitation of particles by photoelectrical method. Registration of concentrations. Problems of mechanics.

[B16] Pedley TJ (1980). The fluid mechanics of large blood vessels.

[B17] Regurer SA (1980). Lectures for biological mechanics.

[B18] Pavlovski UN, Regurer SA, Skobeleva JM (1970). Hydrodynamics of blood.

[B19] Minz DM (1952). About weight of grain layer in rising flow of fluid. Reports RAN.

[B20] Navruzov K, Akhmedzhanova GD, Yakubov BS, Khakberdiev DB (1989). The motion of non Newtonian fluid in tubes with allowing for a wall layer. Academy of Science of Uzbekistan.

[B21] Rakhmatullin KA (1956). Foundations of gas dynamics of mutually penetrable flows of compressible medium. (Russian). Prikladnaya Matematika Mekhanika.

[B22] Batchelor GK (1967). An introduction to fluid dynamics.

[B23] Malinovskaya TA (1971). Separation of suspension in industry of limited synthesis.

[B24] Navruzov KN, Khakberdiev ZB (2000). Dynamics of Non-Newtonian fluids.

[B25] Podrabinek PA, Kamenskii II (1967). On the Gulden size of erythrocytes. Laboratornoye delo.

[B26] Gavalov SM (1957). Mechanism of fractional erythrocyte sedimentation rate. Sov Med.

[B27] Thomas DG (1965). Transport Characteristics of Suspension: VIII. A Note on the Viscosity of Newtonian Suspensions of Uniform Spherical Particles. J Coll Sci.

[B28] Khusanov H (1986). Sedimentation of particles in fluid. Nauka.

[B29] Caro CG (1978). The mechanics of the circulation.

[B30] Faizullaev FD (1969). Laminar motion of multiphase media in conduits.

[B31] Nigmatullin RI (1978). Mechanics of heterogeneous medium.

[B32] Bergentz SE, Gelin LE, Rudenstam CM, Zederfeldt B (1963). The viscosity of whole blood in trauma. Acta Chir Scand.

[B33] Knisely MH, Elliot TW, Block EH (1945). Sludged blood in traumatic shock. Arch Surg.

[B34] Seligman AM, Frank HA, Fine J (1946). Traumatic shock. XII. Hemodynamic effects of alterations of blood viscosity in normal dogs and in dogs in shock. J Clin Invest.

[B35] Tatarishvili J, Momtselidze N, McHedlishvili G (2004). Blood rheological abnormalities in the microcirculation during experimental traumatic shock. Clin Hemorheol Microcirc.

[B36] Heimbeckersh RO, Bigelow WG (1950). Intravascular agglutination of erythrocytes (sludged blood) and traumatic shock. Surgery.

[B37] Mchedlishvili G, Gobejishvili L, Mamaladze A, Momtselidze N, Varazashvili M (1999). Microcirculatory stasis induced by hemorheological disorders: further evidence. Microcirculation.

[B38] Collins AJ (1989). Kidney dialysis treatment for victims of the Armenian earthquake. N Engl J Med.

[B39] Oda J, Tanaka H, Yoshioka T, Iwai A, Yamamura H, Ishikawa K, Matsuoka T, Kuwagata Y, Hiraide A, Shimazu T, Sugimoto H (1997). Analysis of 372 patients with Crush syndrome caused by the Hanshin-Awaji earthquake. J Trauma.

[B40] Shaw AD, Sjolin SU, McQueen MM (1994). Crush syndrome following unconsciousness: need for urgent orthopaedic referral. BMJ.

[B41] Chernysheva GA, Plotnikov MB, Smol'yakova VI (2000). Relationship between rheological and hemodynamic changes in rats with crush syndrome. Bull Exp Biol Med.

[B42] Chernysheva GA, Plotnikov MB, Smol'yakova VI, Aliev OI, Ulanova EV, Sutormina TG (2001). Course of shock in rats with different resistance to shockogenic trauma during Crush syndrome. Bull Exp Biol Med.

[B43] Haugan A, Kirkebo A (1984). Local blood flow changes in the renal cortex during tourniquet and burn shock in rats. Circ Shock.

[B44] Cryer HG, Mavroudis C, Yu J, Roberts AM, Cue JI, Richardson JD, Polk HC (1990). Shock, transfusion, and pneumonectomy. Death is due to right heart failure and increased pulmonary vascular resistance. Ann Surg.

[B45] Carleton SC (1995). Cardiac problems associated with burns. Cardiol Clin.

[B46] Ismailov RM, Shevchuk NA, Schwerha J, Keller L, Khusanov H (2004). Blunt trauma to large vessels: a mathematical study. Biomed Eng Online.

[B47] Ismailov RM (2005). Mathematical model of blunt injury to the vascular wall via formation of rouleaux and changes in local hemodynamic and rheological factors. Implications for the mechanism of traumatic myocardial infarction. Theor Biol Med Model.

